# Modeling diurnal Temperature-Rainfall relationships under multicollinearity using PLS-SEM: A case study of Ghana

**DOI:** 10.1371/journal.pone.0351942

**Published:** 2026-06-30

**Authors:** Isaac Osei, Anil Carie, Acheampong Baafi-Adomako, Dennis Opoku Boadu

**Affiliations:** 1 Department of Computer Science and Engineering, SRM University-AP, Amaravati, Andhra Pradesh, India; 2 Department of Research and Applied Meteorology, Ghana Meteorological Agency, Accra, Ghana; 3 Department of Computer Science, University of Ghana, Legon, Accra, Ghana; The Chinese University of Hong Kong, HONG KONG

## Abstract

Rainfall variability in tropical climates is influenced by interacting thermal processes, yet maximum (TMAX) and minimum (TMIN) temperatures are often highly collinear, complicating the estimation of their distinct relationships with precipitation. This study applies Partial Least Squares Structural Equation Modeling (PLS-SEM) to long-term station-based climate data from Ghana (1981–2020; N = 252 station × calendar month observations) to examine structural associations among TMAX, TMIN, and rainfall (RAIN), including differences between coastal and inland regimes. The model represents a simplified component of the broader hydroclimatic system. The results indicate a statistically significant negative association between TMAX and rainfall (*β* = −0.454, p < 0.001) and a positive association between TMIN and rainfall (*β* = 0.166, p < 0.001). The relationship between TMAX and TMIN is positive but not statistically significant (*β* = 0.152, p = 0.053). Mediation analysis does not support a significant indirect pathway from TMAX to rainfall via TMIN (*β* = 0.025, p = 0.076). The model explains 21.1% of rainfall variance. Multi-group analysis reveals spatial heterogeneity, with a stronger negative TMAX-rainfall association inland (*β* = −0.721) than along the coast (*β* = −0.427), and a stronger TMAX-TMIN association in coastal regions (*β* = 0.753 vs. 0.337). The findings suggest that TMAX and TMIN exhibit distinct statistical relationships with rainfall that vary across climatic regimes. However, given the limited variables and observational design, the results should be interpreted as structural associations rather than definitive physical mechanisms. The study demonstrates the utility of PLS-SEM for handling multicollinearity and interdependent relationships in climate data while highlighting the need to incorporate additional atmospheric variables to improve explanatory depth.

## 1. Introduction

Rainfall variability in tropical regions arises from complex interactions among thermal, dynamical, and moisture-related processes operating across diurnal to seasonal timescales [1]. In West Africa, and particularly in Ghana, relatively small variations in temperature are often associated with substantial changes in precipitation patterns, with important implications for agriculture, water resources, energy systems, and climate risk management [2–4]. Understanding how temperature variability relates to rainfall is therefore an important component of regional climate analysis [5,6]. However, rainfall in tropical systems is influenced by a wide range of processes, including atmospheric moisture availability, circulation dynamics, and large-scale climate drivers, of which temperature represents only one component.

A growing body of literature highlights the relevance of diurnal temperature dynamics for precipitation processes. Maximum temperature (TMAX) reflects daytime surface heating and energy availability for convection, whereas minimum temperature (TMIN) captures nighttime radiative balance, boundary-layer stability, and cloud persistence [7–9]. These temperature extremes are typically interrelated, forming a coupled thermal system in which daytime conditions influence nocturnal states. Rainfall emerges from this broader system through nonlinear interactions involving atmospheric instability, moisture processes, and cloud dynamics [10–12].

Despite this physical understanding, empirical analyses often treat TMAX and TMIN as independent predictors within conventional regression frameworks, without explicitly accounting for their interdependence or potential indirect relationships with rainfall [13,14]. A key challenge in this context is multicollinearity: TMAX and TMIN are frequently highly correlated, which can lead to unstable parameter estimates and reduced interpretability in standard regression models [15]. While alternative approaches such as ridge regression can address collinearity, they do not explicitly model interdependent relationships among variables.

Structural equation modeling (SEM) provides a framework for simultaneously estimating multiple relationships, including direct and indirect pathways, within a unified system [16,17]. In this study, we employ Partial Least Squares Structural Equation Modeling (PLS-SEM), a variance-based approach that is robust to multicollinearity and does not rely on strict distributional assumptions [18–20]. The use of PLS-SEM in this context is therefore motivated by its ability to estimate interdependent relationships under collinearity, rather than by latent variable modeling per se.

While PLS-SEM has been widely applied in social sciences [21] and environmental research [22], its application in atmospheric and climatological studies remains limited. This study contributes to this emerging area by applying PLS-SEM to long-term station-based climate data from Ghana (1981–2020) to examine the relationships among TMAX, TMIN, and rainfall. Importantly, the model is intentionally parsimonious and focuses on thermal variables; it is therefore not intended to represent the full set of processes governing rainfall variability.

The study is guided by the following research questions:

**RQ1:** *How are maximum temperature (TMAX) and minimum temperature (TMIN) statistically associated with rainfall variability in Ghana?*

**RQ2:** *Do these associations differ between coastal and inland climate regimes?*

By addressing these questions, the study makes two primary contributions. First, it provides a methodological contribution by demonstrating how PLS-SEM can be used as a complementary approach for analyzing multicollinearity and interdependent relationships in climate datasets. Second, it offers an empirical contribution by characterizing the statistical associations between diurnal temperature measures and rainfall across different climatic regimes in Ghana. The findings are intended to complement, rather than replace, physically based climate modeling approaches.

The remainder of the paper is structured as follows. Section 2 synthesizes the relevant literature and develops the conceptual framework; Section 3 describes the data and methodology; Sections 4 and 5 present and discuss the results; and Section 6 concludes.

## 2. Literature review and hypotheses development

### 2.1 Diurnal temperature dynamics

Diurnal temperature variability is a fundamental characteristic of the climate system, reflecting the balance between daytime solar heating and nighttime radiative cooling [23,24]. Maximum temperature is primarily controlled by surface energy fluxes, cloud cover, and atmospheric circulation, whereas minimum temperature is influenced by nocturnal longwave radiation, humidity, and boundary-layer processes [7–9]. Empirical evidence consistently demonstrates strong persistence between daytime and nighttime temperatures, particularly in tropical and subtropical regions [24,25].

Within a statistical modeling framework, this thermal coupling can be represented as a directional association from TMAX to TMIN, reflecting the tendency for daytime conditions to be related to subsequent nighttime thermal states. Accordingly, the following hypothesis is formulated:

**H1:** *TMAX is positively associated with TMIN.*

### 2.2 Temperature-Rainfall relationships

Temperature plays a central role in precipitation processes by shaping atmospheric instability, moisture-holding capacity, and convective potential. Observational scaling studies demonstrate that temperature increases modify the precipitation-moisture relationship through changes in convective available potential energy (CAPE) and thermodynamic constraints [26]. Higher daytime temperatures can enhance CAPE and promote rainfall under moisture-sufficient conditions, while suppressing precipitation in moisture-limited regimes by increasing atmospheric moisture demand [27–29]. Minimum temperature, by contrast, characterizes nighttime boundary-layer conditions and condensation efficiency, influencing rainfall (RAIN) persistence and intensity through its association with atmospheric stability and cloud maintenance processes [1,23,30,31].

While these processes are well established in the climatological literature, the present study focuses on estimating statistical relationships among observed variables. Accordingly, the following hypotheses are proposed:

**H2:** *TMAX is significantly associated with RAIN.*

**H3:** *TMIN is significantly associated with RAIN.*

### 2.3 Mediation of diurnal thermal effects

Climatic influences often operate through interconnected processes spanning diurnal cycles and linked atmospheric states. In the context of diurnal temperature dynamics, daytime heating (TMAX) influences boundary-layer development, atmospheric moisture demand, and humidity conditions, which can in turn be associated with nighttime thermal states (TMIN) and subsequent precipitation-related processes [32–34]. Empirical evidence further indicates that daytime warming is associated with increased atmospheric dryness and vapor pressure deficit, with TMAX exerting a stronger influence on dryness than TMIN over interannual scales [30]. This implies that daytime warming can indirectly modify nighttime thermal states and associated moisture processes relevant to rainfall outcomes. Recent studies on sub-daily thermal perturbations further show that thermal variability across diurnal cycles can influence precipitation by modulating atmospheric instability and moisture convergence [35]. In addition, prior climatological research suggests that minimum temperature may play a role in transmitting part of the influence of maximum temperature to precipitation through its association with boundary-layer stability and condensation processes [36].

These findings suggest that part of the association between TMAX and rainfall may operate indirectly through its relationship with TMIN. However, given the observational nature of the data and the simplified model specification, the mediated pathway is interpreted as a statistical representation of associations rather than definitive evidence of physical transmission mechanisms. Its inclusion is intended to assess whether incorporating TMIN alters the estimated relationship between TMAX and RAIN.

Accordingly, we propose:

**H4:** *TMIN statistically mediates the association between TMAX and RAIN.*

### 2.4 Moderation by coastal-inland climate regimes

Climate-rainfall relationships are expected to vary across spatial regimes due to differences in moisture availability, land-sea interactions, and boundary-layer processes [37,38]. Coastal environments are typically characterized by persistent maritime moisture supply, moderated temperature variability, and enhanced atmospheric humidity, which can reduce temperature constraints on rainfall formation [38,39]. In contrast, inland regions are more strongly influenced by continental heating, greater diurnal temperature ranges, and more moisture-limited conditions, where temperature variability may be more strongly associated with precipitation dynamics [30,37].

These differences suggest that the statistical associations among TMAX, TMIN, and rainfall may not be spatially uniform, but instead vary systematically across coastal and inland regimes. In this study, coastal-inland classification is used as a grouping variable to examine such heterogeneity.

Accordingly, the following hypothesis is proposed:

**H5:** *The statistical relationships among TMAX, TMIN, and RAIN differ between coastal and inland climate regimes.*

**Fig 1:**
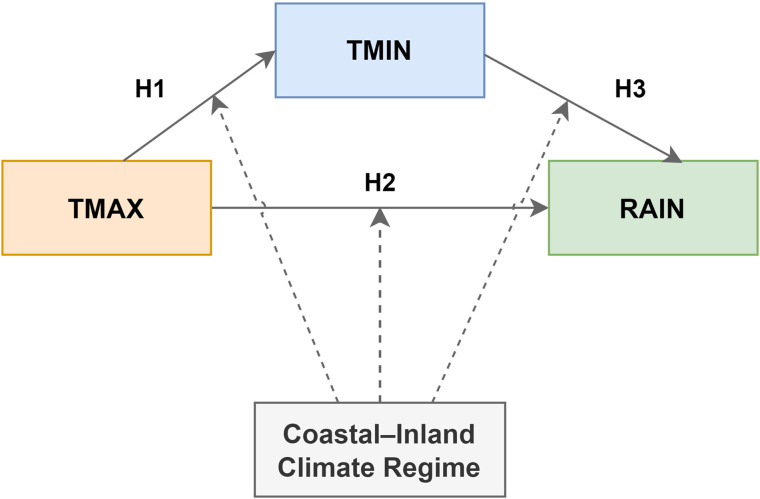
Conceptual framework of the proposed research model. Source: Authors’ own illustration.

Based on these relationships, Fig 1 presents the conceptual model examined in this study.

### 2.5 PLS-SEM in climatological research

Partial least squares structural equation modeling (PLS-SEM) is a flexible multivariate technique that combines path modeling, factor analysis, regression analysis, and latent variable estimation [19,40,41]. Traditionally, PLS-SEM has been widely applied in social science and management research [20,21,42,43]. However, its application in environmental and climate-related studies has grown in recent years due to its robustness to multicollinearity, non-normal data distributions, and flexibility across different sample sizes [18,19].

A standard PLS-SEM framework comprises two interconnected components: the measurement model and the structural model [44,45]. The measurement model defines relationships between latent variables and their observed indicators, enabling the assessment of indicator reliability and construct validity. The structural model specifies the hypothesized relationships among variables, allowing the estimation of direct, indirect, and conditional associations within complex systems.

In the present study, the variables are directly observed climate measures rather than latent constructs with multiple indicators. As such, the model can be interpreted as a path-analytic specification estimated using the PLS-SEM algorithm. The choice of PLS-SEM is motivated primarily by its ability to estimate interdependent relationships under conditions of multicollinearity, rather than by latent variable modeling. In this sense, PLS-SEM is used here as a variance-based structural modeling approach that accommodates collinearity while allowing simultaneous estimation of multiple relationships.

Recent applications of SEM have been reported in ecological systems [22,46], hydrology [40,47], and climate vulnerability assessments [48,49]. Despite this progress, the use of SEM, particularly PLS-SEM, in atmospheric and climatological research remains limited. This is notable given that climate datasets often exhibit strong multicollinearity (e.g., between minimum and maximum temperature), multidimensional relationships, and measurement uncertainty.

Compared with conventional regression-based approaches, time-series models, or penalized methods such as ridge regression, PLS-SEM provides a flexible framework for modeling interdependent relationships, including mediation and group-specific effects, within a unified system. Accordingly, this study adopts PLS-SEM as a complementary methodological approach for examining temperature-rainfall relationships under conditions of multicollinearity.

## 3. Methodology

### 3.1 Data source and preparation

Daily station-level climate data for the period 1981–2020 were obtained from the Ghana Meteorological Agency (GMet). The dataset includes observations of minimum temperature (°C), maximum temperature (°C), and rainfall (mm) from 21 meteorological stations representing Ghana’s major climatic zones. Standard quality-control procedures were applied, including screening for outliers, internal consistency checks, and treatment of missing values.

**Fig 2:**
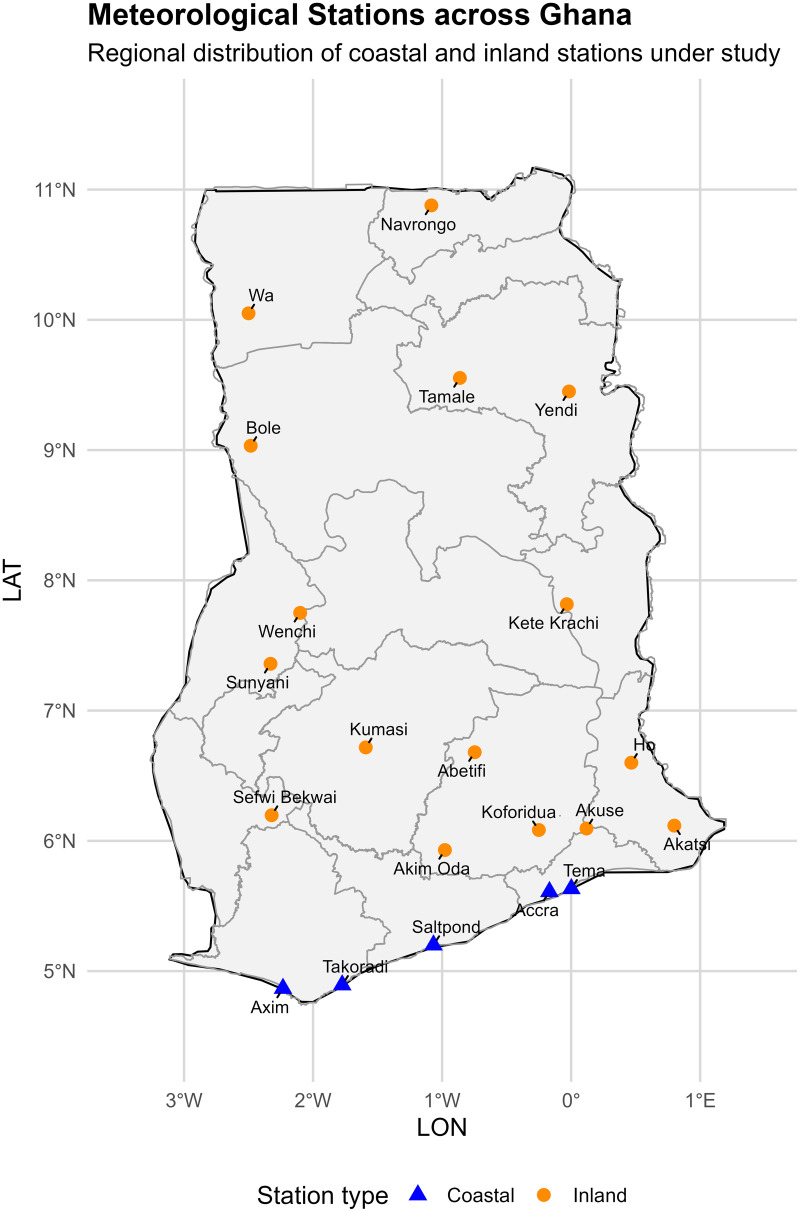
Spatial distribution of the meteorological stations used in this study across Ghana. Source: Authors’ plot in R using geodata for Ghana.

To ensure temporal comparability and reduce high-frequency noise, daily observations were first aggregated into monthly values for each station. Fig 2 presents the spatial distribution of the meteorological stations used in this study, plotted in **R** [50] (version 4.5.1) using the **ggplot2** package [51].

The unit of analysis in the final dataset is defined as station × calendar month. For each station (n = 21) and each month of the year (12 months), long-term climatic averages were computed, yielding a total of: *N* = *21* × *12* = *252* observations

Specifically, monthly values were first summarized within four decadal periods (1981–1990, 1991–2000, 2001–2010, and 2011–2020). For each station and calendar month (e.g., all Januaries within a decade), decadal averages were computed and subsequently averaged across the four periods to obtain a single long-term monthly climatology for each station.

This aggregation strategy (daily → monthly → decadal → long-term monthly composite) reduces short-term variability while preserving systematic seasonal and spatial structure. The resulting dataset captures typical monthly climatic conditions rather than interannual variability and should therefore be interpreted as a set of climatological summaries rather than independent time-series realizations. Accordingly, the analysis focuses on estimating structural associations among variables rather than making causal or predictive claims about temporal dynamics.

To enable spatial comparison, stations were classified into coastal and inland groups based on the operational classification provided by the GMet. This classification reflects established climatological zoning used in national meteorological analyses and is consistent with prior studies distinguishing coastal and inland regimes in Ghana [52–54]. Based on this categorization, the dataset comprises 5 coastal stations and 16 inland stations, corresponding to 60 and 192 observations, respectively. Differences in station density and spatial coverage between climatic regions may influence the stability and representativeness of regional climate analyses, particularly when comparing heterogeneous climatic regimes [55]. Accordingly, the multi-group results should be interpreted with appropriate caution.

We assessed sample size adequacy for PLS-SEM using G*Power [56] (version 3.1.9.7). Following established guidelines, a small-to-moderate effect size (f² = 0.08), a significance level of 0.05, statistical power of 0.95, and two predictors were specified [19,57,58]. The analysis indicated a minimum required sample size of 165 observations, which is below the available sample (*N* = 252).

### 3.2 Measurement model specification

Decadal climate indicators were aggregated into long-term composite indices using arithmetic means. These composites represent average climatological conditions for each station-month combination and were used as single-indicator constructs in the model.

The use of single-indicator constructs is appropriate because the variables (TMAX, TMIN, and RAIN) are directly observed physical measurements rather than latent constructs.

Given the instrumental nature of the data, measurement error is assumed to be minimal. As a result, explicit measurement model evaluation (e.g., composite reliability, AVE) is not applicable.

### 3.3 Structural model and estimation

The structural model specifies direct paths from TMAX to TMIN and RAIN, and from TMIN to RAIN, as well as an indirect path from TMAX to RAIN via TMIN.

Model estimation was conducted using the PLS algorithm implemented in the **seminr** package [59] (version 2.3.7) in **R**. Bootstrapping with 10,000 resamples was used to assess the statistical significance of path coefficients and indirect effects.

The structural relationships were formalized as:


$$\begin{array}{c} 𝐓𝐌𝐈𝐍\;=\;\beta_{\text{1}}.TMAX+\zeta_{\text{1}} \end{array}$$ (1)

$$\begin{array}{c} 𝐑𝐀𝐈𝐍\;=\;\beta_{\text{2}}.TMAX+\beta_{\text{3}}.TMIN+\zeta_{\text{2}} \end{array}$$ (2)

where β_1_, β_2_, and β_3_ are standardized path coefficients, and ζ_1_ and ζ_2_ represent disturbance terms capturing unexplained variance.

The indirect (mediated) effect of TMAX on rainfall is computed as:


$$\begin{array}{c} 𝐈𝐧𝐝𝐢𝐫𝐞𝐜𝐭\;𝐄𝐟𝐟𝐞𝐜𝐭\;=\;\beta_{\text{1}}\times \;\beta_{\text{3}} \end{array}$$ (3)

Given the observational and aggregated nature of the data, mediation is interpreted as a statistical decomposition of associations, rather than evidence of a physical transmission mechanism.

To examine spatial heterogeneity, a multi-group analysis (MGA) was conducted comparing coastal and inland stations. The PLS-MGA procedure implemented in **seminr** was used to assess differences in path coefficients between groups using bootstrapped estimates. This non-parametric approach avoids distributional assumptions and is suitable for comparing structural relationships across groups. Results should be interpreted as indicative of relative differences in associations, given potential differences in group sizes and spatial coverage.

## 4. Results

### 4.1 Descriptive statistics

Table 1 summarizes the descriptive statistics of the composite climate variables used in the PLS-SEM analysis (N = 252). Mean maximum temperature (TMAX) is 32.05°C (SD = 2.64), while mean minimum temperature (TMIN) is 22.99°C (SD = 1.50), indicating moderate thermal variability across stations and climatological monthly units. Rainfall exhibits substantially greater dispersion (SD = 73.41 mm), with values ranging from near-zero precipitation (0.13 mm) to high monthly totals (493.14 mm).

**Table 1:** Descriptive statistics of composite climate variables (1981–2020; N = 252).

Variable	N	Mean	SD	Minimum	Maximum
TMAX (°C)	252	32.05	2.64	26.44	39.93
TMIN (°C)	252	22.99	1.5	17.34	26.51
RAIN (mm)	252	97.89	73.41	0.13	493.14

*Note: N = Total number of observations; SD = Standard deviation (Source: Authors’ own compilation)*.

The comparatively high variability in rainfall relative to temperature reflects pronounced spatial and seasonal heterogeneity in moisture availability across Ghana’s climatic zones. Given that the dataset represents long-term climatological averages (station × month), this variability captures systematic spatial and seasonal differences rather than short-term extremes. This supports subsequent analysis of statistical associations among variables and potential regime-specific differences.

### 4.2 Structural model results

**Fig 3:**
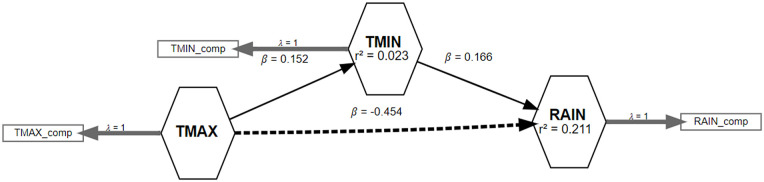
Measurement and structural model results. Source: Authors’ output from RStudio.

The structural model results are summarized in Table 2 and illustrated in Fig 3.

**Table 2:** Structural model path coefficients and bootstrapping results.

H	Path	β	R^2^	f^2^	SE	T Stat.	95% CI	p-value	Supp.
H1	TMAX → TMIN	0.152	0.023	0.024	0.079	1.936	[0.000, 0.308]	0.053	No
H2	TMAX → RAIN	−0.454	0.211	0.256	0.044	−10.427	[-0.538, -0.368]	< 0.001	Yes
H3	TMIN → RAIN	0.166	0.211	0.034	0.045	3.686	[0.075, 0.251]	< 0.001	Yes

*Note: H = Hypothesis; β = Path coefficient; R*^*2*^ *= Explanatory power; f*^*2*^ *= Effect size; SE = Standard error; T Stat. = Test statistics; CI = Confidence interval; Supp. = Supported (Source: Authors’ own compilation)*.

H1 predicted a positive association between maximum and minimum temperature. Although the estimated path coefficient is positive (*β* = 0.152), the effect is marginal but does not reach conventional statistical significance at the 5% level (t = 1.936, p = 0.053). Therefore, H1 is not supported. H2 proposed a significant association between maximum temperature and rainfall. The results indicate a statistically significant negative relationship (*β* = −0.454, p < 0.001), suggesting that higher daytime temperatures are associated with lower rainfall levels within the aggregated climatological dataset. H3 predicted a significant association between minimum temperature and rainfall. This hypothesis is supported (*β* = 0.166, p < 0.001), indicating that higher nighttime temperatures are positively associated with rainfall. The model explains 2.3% (R² = 0.023) of the variance in minimum temperature and 21.1% (R² = 0.211) of the variance in rainfall. While this level of explanatory power is moderate, it indicates that a substantial proportion of rainfall variability remains unexplained, consistent with the multifactorial nature of precipitation processes.

### 4.3 Mediation analysis

Bootstrapped mediation results are presented in Table 3. H4 proposed that minimum temperature mediates the association between maximum temperature and rainfall. The indirect effect of TMAX on RAIN via TMIN is positive but not statistically significant (*β* = 0.025, t = 1.774, p = 0.076), with a 95% confidence interval [0.000, 0.056] that includes zero. Therefore, H4 is not supported.

**Table 3:** Indirect effect results.

Path	β	SE	T Stat.	95% CI	p-value	Supp.
TMAX → TMIN → RAIN	0.025	0.014	1.774	[0.000, 0.056]	0.076	No

*Note: β = Path coefficient; SE = Standard error; T Stat. = Test statistics; CI = Confidence interval; Supp. = Supported (Source: Authors’ own compilation)*.

This finding indicates that, within the specified model, the association between maximum temperature and rainfall is not meaningfully transmitted through minimum temperature. Instead, the relationship is primarily captured by the direct path from TMAX to RAIN.

### 4.4 Multi-group (regime) analysis

The PLS-MGA results are reported in Table 4. H5 predicted that the relationships among TMAX, TMIN, and RAIN differ between coastal and inland climate regimes. The results indicate statistically significant differences for two structural paths. First, the negative association between maximum temperature and rainfall is significantly stronger in inland regions (*β* = −0.721) than in coastal regions (*β* = −0.427), with a significant difference (Δ*β* = 0.293, p = 0.027). This suggests that rainfall in inland areas is more strongly associated with variations in daytime temperature. Second, the association between maximum and minimum temperature is significantly stronger in coastal regions (*β* = 0.753) compared to inland regions (*β* = 0.337), with a highly significant difference (Δ*β* = 0.416, p < 0.001), indicating stronger diurnal thermal coupling in coastal environments. No statistically significant difference is observed for the TMIN → RAIN relationship (p = 0.802), suggesting that the association between nighttime temperature and rainfall is relatively consistent across regimes.

**Table 4:** PLS-MGA results.

Path	Coastal β	Inland β	Δβ	p-value	Significant
TMAX → RAIN	−0.427	−0.721	0.293	0.027	Yes
TMIN → RAIN	0.311	0.416	−0.105	0.802	No
TMAX → TMIN	0.753	0.337	0.416	< 0.001	Yes

*Note: β = Path coefficient; Δ = Change (Source: Authors’ own compilation)*.

Overall, these findings support H5 and indicate spatial heterogeneity in the estimated relationships. However, differences in group sizes and spatial coverage should be considered when interpreting these results.

## 5. Discussion

The results provide empirical evidence that rainfall variability in Ghana is associated with interacting thermal processes rather than isolated temperature effects. While maximum and minimum temperatures are positively related, the overall coupling between TMAX and TMIN is modest in the full sample and does not reach conventional statistical significance. However, the multi-group analysis indicates that this association is substantially stronger in coastal regions, suggesting that maritime influence is linked with greater diurnal thermal continuity.

Contrary to a simple heating-precipitation trigger interpretation, maximum temperature (TMAX) exhibits a strong and statistically significant negative association with rainfall. This finding suggests that elevated daytime heating is linked with reduced rainfall levels within the aggregated climatological dataset. One plausible explanation, consistent with prior studies, is that higher daytime temperatures may be associated with enhanced evapotranspiration and boundary-layer drying under moisture-limited inland conditions, which can reduce effective precipitation (e.g., [54,60–62]). However, given the simplified model and absence of explicit moisture or circulation variables, this interpretation should be viewed as consistent with, rather than definitive evidence of, underlying physical processes.

Minimum temperature (TMIN), in contrast, shows a significant positive association with rainfall. This relationship aligns with climatological evidence indicating that warmer nocturnal conditions are often associated with higher atmospheric moisture and conditions favorable for sustained precipitation. Tropically focused analyses demonstrate that diurnal cycles of atmospheric water vapor and cloud development are closely linked to precipitation processes, particularly in moisture-rich environments [23]. Observational studies further show that increases in minimum temperature are associated with higher relative humidity and atmospheric moisture availability, which can support rainfall persistence [63]. In addition, humid heatwave events, often characterized by elevated nighttime temperatures, have been linked to increased probabilities of extreme precipitation [64]. Long-term trend analyses also document coherent patterns between TMIN and precipitation variability across climatic zones [65]. Taken together, these findings are consistent with the positive TMIN-RAIN association observed in this study.

Importantly, the mediation analysis indicates that the indirect pathway TMAX → TMIN → RAIN is not statistically significant. This indicates that the association between maximum temperature and rainfall is not meaningfully transmitted through minimum temperature. Instead, daytime and nighttime temperature variables appear to exhibit partially independent associations with rainfall. In addition, the observed associations may partly reflect reciprocal hydroclimatic relationships, in which rainfall variability is often associated with changes in surface temperature through processes such as evaporative cooling, cloud persistence, and radiative effects.

The multi-group analysis further indicates that temperature-rainfall relationships vary across climatic regimes. The negative association between TMAX and rainfall is significantly stronger in inland regions, whereas the association between TMAX and TMIN is stronger in coastal zones. These patterns are consistent with known hydroclimatic contrasts across West Africa. Coastal environments are influenced by maritime moisture supply and ocean-atmosphere interactions that moderate temperature variability and support moisture availability, while inland regions are more strongly affected by continental heating and moisture limitations. Previous observational and reanalysis studies document similar contrasts, with coastal precipitation linked to oceanic moisture fluxes and inland rainfall more sensitive to land-atmosphere interactions and monsoon dynamics [66–68]. The present results are therefore consistent with these broader regional patterns, while highlighting how such differences are reflected in statistical associations among temperature variables and rainfall.

From a methodological perspective, this study demonstrates the usefulness of PLS-SEM for analyzing interdependent climatic variables in the presence of multicollinearity. Climate variables often exhibit strong intercorrelations and simultaneous relationships, which can complicate interpretation in conventional regression frameworks. The PLS-SEM approach provides a flexible way to estimate such interdependencies, including direct and indirect associations, while remaining robust to collinearity and distributional constraints [19,43,57,69].

Overall, the findings highlight three key insights. First, higher daytime temperatures are associated with reduced rainfall, particularly in inland regions. Second, nighttime temperature exhibits a positive association with rainfall, suggesting a distinct role in hydroclimatic variability. Third, these relationships are not spatially uniform but vary across coastal and inland regimes.

These results contribute to a more nuanced understanding of Ghana’s hydroclimatic system by emphasizing the importance of considering interacting temperature variables and spatial heterogeneity. At the same time, the findings should be interpreted in light of the study’s simplified model structure and aggregated data.

## 6. Conclusion

This study examined the structural relationships among maximum temperature, minimum temperature, and rainfall in Ghana using Partial Least Squares Structural Equation Modeling (PLS-SEM), with particular attention to diurnal thermal dynamics and regional heterogeneity. The analysis was motivated by the need to move beyond isolated temperature-rainfall correlations toward a more integrated assessment of how interacting thermal variables are associated with precipitation variability in tropical environments. Given the strong interdependence among climate variables and ongoing hydroclimatic variability in West Africa, assessing both direct associations and regime-specific differences is essential.

The findings indicate that temperature-rainfall relationships are not uniform and vary across climatic regimes. Maximum temperature exhibits a statistically significant negative association with rainfall, with this relationship being stronger in inland regions. Minimum temperature shows a positive and significant association with rainfall. Although TMAX and TMIN are positively related, the indirect pathway is not statistically significant, suggesting that the associations of daytime and nighttime temperatures with rainfall operate largely independently within the specified model. In addition, the stronger association between TMAX and TMIN observed in coastal regions suggests that temperature relationships vary across spatial regimes.

These findings have several implications. From a climatological perspective, they highlight the importance of considering diurnal temperature structure when examining rainfall variability, particularly in tropical systems characterized by strong land-atmosphere and moisture interactions. The observed inland sensitivity of rainfall to maximum temperature suggests that temperature variability may play a more pronounced role in shaping rainfall patterns in moisture-limited environments. From a methodological standpoint, the study demonstrates that PLS-SEM can be used as a flexible framework for modeling interdependent climate variables under conditions of multicollinearity, enabling the estimation of direct and indirect associations as well as group-specific differences within a unified structure.

However, the results should be interpreted in light of several limitations. The model includes a limited set of variables and does not explicitly account for atmospheric moisture, circulation dynamics, or large-scale climate drivers known to influence rainfall. The use of aggregated climatological averages (station × month) reduces short-term variability and implies that the findings reflect long-term statistical associations rather than temporal dynamics or causal processes. In addition, spatial heterogeneity was represented using a coastal-inland grouping, which provides a simplified characterization of regional variability and may be influenced by differences in station density and spatial coverage.

Future research could extend this work by incorporating additional hydroclimatic variables such as relative humidity, precipitable water, and circulation indices, as well as by exploring alternative modeling approaches, including spatially explicit or hybrid statistical-process-based frameworks. Such extensions would help to better capture the complexity of rainfall processes and improve the interpretability and predictive relevance of the results.

Overall, this study contributes to the understanding of Ghana’s hydroclimatic system by showing that rainfall variability is associated with interacting diurnal temperature characteristics that vary across spatial regimes. The findings provide a useful basis for further investigation into coupled temperature-rainfall dynamics in tropical climates.
